# Pioglitazone-Loaded PLGA Nanoparticles: Towards the Most Reliable Synthesis Method

**DOI:** 10.3390/ijms23052522

**Published:** 2022-02-25

**Authors:** Biagio Todaro, Aldo Moscardini, Stefano Luin

**Affiliations:** 1 National Enterprise for NanoScience and NanoTechnology (NEST) Laboratory, Scuola Normale Superiore, Piazza San Silvestro 12, I-56127 Pisa, Italy; aldo.moscardini@sns.it; 2 National Enterprise for NanoScience and NanoTechnology (NEST) Laboratory, Istituto Nanoscienze, CNR, Piazza San Silvestro 12, I-56127 Pisa, Italy

**Keywords:** pioglitazone, PLGA, polymeric nanoparticles synthesis, nanoprecipitation, single emulsification-solvent evaporation, encapsulation efficiency, drug loading, drug release kinetics

## Abstract

Recent findings have proved the benefits of Pioglitazone (PGZ) against atherosclerosis and type 2 diabetes. Since the systematic and controllable release of this drug is of significant importance, encapsulation of this drug in nanoparticles (NPs) can minimize uncontrolled issues. In this context, drug delivery approaches based on several poly(lactic-co-glycolic acid) (PLGA) nanoparticles have been rising in popularity due to their promising capabilities. However, a fully reliable and reproducible synthetic methodology is still lacking. In this work, we present a rational optimization of the most critical formulation parameters for the production of PGZ-loaded PLGA NPs by the single emulsification-solvent evaporation or nanoprecipitation methods. We examined the influence of several variables (e.g., component concentrations, phases ratio, injection flux rate) on the synthesis of the PGZ-NPs. In addition, a comparison of these synthetic methodologies in terms of nanoparticle size, polydispersity index (PDI), zeta potential (ζ_p_), drug loading (DL%), entrapment efficiency (EE%), and stability is offered. According to the higher entrapment efficiency content, enhanced storage time and suitable particle size, the nanoprecipitation approach appears to be the simplest, most rapid and most reliable synthetic pathway for these drug nanocarriers, and we demonstrated a very slow drug release in PBS for the best formulation obtained by this synthesis.

## 1. Introduction

Atherosclerosis is a heavy condition characterized by progressive inflammation and slowly calcifying lesions in the intima and inner media of the arterial wall due to plaque formation [1]. The incidence of this disease is rising worldwide. Several millions of people were affected with atherosclerosis in 2021, and its burden is continuously rising, which may lead to an enormous effect on both the world economy and manpower [2]. Furthermore, atherosclerotic lesions are worsened in type-2 diabetes. Recent studies have reported that people with diabetes are more likely to have a carotid plaque with calcification and lipid-rich necrotic cores than people without diabetes [3,4].

Albeit, the relationship between diabetes drugs and the progression of atherosclerosis is still elusive, Pioglitazone (PGZ; 5-[[4-[2-(5-ethylpyridin-2-yl)ethoxy]phenyl]methyl]–1,3- thiazolidine-2,4-diona), one of the most frequently prescribed anti-diabetic medication in the United States, slows the progression of atherosclerosis [5]. PGZ is a slightly hydrophobic small molecule (logP = 2.3; experimental value from Human Metabolome Database) commonly used in treatment, or progression control, of type 2 diabetes. This drug acts by principally stimulating the nuclear receptor peroxisome proliferator-activated receptor gamma (PPAR-γ), increasing, therefore, the sensitivity of peripheral tissues to insulin, reducing gluconeogenesis resistance in the liver and finally inhibiting macrophage activation and atherosclerotic plaque ruptures [6,7]. However, the application of PGZ is seriously limited due to its low and pH-dependent solubility, low half-life in plasma due to rapid liver metabolism, dose-dependent systemic side effects and nonspecific drug delivery [8]. To overcome this problem, suitable nanocarriers, such as nanoparticles (NPs), can improve PGZ therapeutic efficacy on PPAR-γ and reduce its side effects [9,10].

Nanoparticles are nanosystems in which a synthetic or natural polymeric membrane delimits a cavity that incorporates the active substance (nanocapsules), or in which the active substance is uniformly dispersed (nanospheres) [11]. In the broad sense of the term, NPs drug delivery approach relies upon the possibility of carrying active molecules mostly to the diseased tissue of interest and with a higher intracellular uptake than free drugs [12]. This elicits not only a significant drug protection from systemic degradations, but also a greater safeguard of healthy tissues [13]. In nanomedicine, several types of NPs have been widely investigated [14,15]. Various nanoscale colloidal carriers, such as carbon nanotubes, dendrimers, inorganic nanoparticles, lipid solid nanoparticles, liposomes, hydrogel nanoparticles and polymeric micelles, were developed and addressed to different therapeutic targets. In polymeric NPs, the polymer is often composed of natural or synthetic monomers; examples of polymers are polyamides, polyanhydrides, polycaprolactone (PLC), polyorthoesters and polyesters, including polylactic acid (PLA), polyglycolic acid (PGA), and poly(lactic-co-glycolic acid) (PLGA) [16,17]. Recent research on PLGA-based NPs for drug delivery is based on the field’s increasing understanding of PLGA properties and procedures of chemical modification, which are applied to the optimization of nanoparticle drug loading and release features. The polymeric NPs degradation’s kinetics are regulated by several factors: (i) the polymer–water interaction, since the more hydrophilic the material, the faster the rate of decomposition; (ii) the polymer crystallinity: the lower the material crystallinity, the faster the degradation; (iii) temperature, since it drives the kinetics of the reaction; (iv) the presence of heteroatoms and/or hydrophilic groups, which makes degradation easier; (v) polymer chain branching and use of initiators in synthesis; (vi) polymer concentration, pH, and salt concentration; (vii) in the case of PLGA, GA/LA ratio: it has been shown that optimal stability of the polymer in biofluids is obtained with a ratio of 50/50 LA/GA [18]. PLGA is generally decomposed by hydrolysis, through which oligomers are decomposed into their biocompatible monomers; then, lactic acid (LA) is disposed through the kidneys, like the one produced during intense physical activity in the muscles, whilst glycolic acid (GA) is transformed into pyruvate, which enters into the Krebs cycle [19]. Thus, PLGA is a Food and Drugs Administration (FDA) and European Medicine Agency (EMA)-approved material and is particularly appropriate for drug delivery, with its low toxicity, biocompatibility, biodegradability, and tunable physical properties [20,21].

Based on these advantages, some research was focused on the preparation of PGZ-loaded PLGA nanoparticles [22,23,24,25]. The influence of formulation parameters using different techniques, such as emulsion solvent evaporation/extraction, salting-out, nanoprecipitation, membrane emulsification, microfluidic technology, and flow focusing, was reported [26,27].

**Figure 1:**
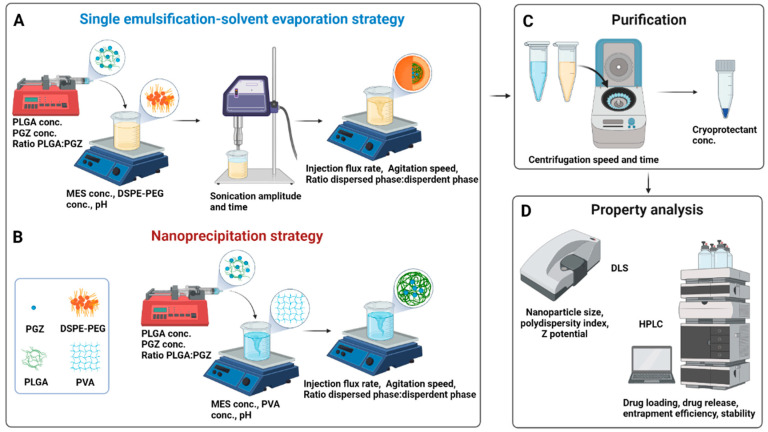
Synthetic workflow for the Pioglitazone-loaded PLGA nanoparticles preparation via single emulsification-solvent evaporation (**A**) or nanoprecipitation (**B**) techniques. A bubble-free non-water miscible (for **A**) or water-miscible (for **B**) organic solution, containing the drug and the polymer, was loaded into a syringe and injected within an aqueous solution (buffer). Nanoparticles formation needs a high shear force in the emulsification approach, while it is spontaneous in the nanoprecipitation approach thanks to phase separation. The organic solvent is then evaporated, and the nanoparticles are washed by centrifugation cycles and resuspended in a cryoprotectant solution for storage at low temperatures (**C**). Parameters that can be tuned are indicated within the panels (**A**–**C**). Physicochemical properties of nanoparticles are finally investigated through DLS and HPLC (**D**). Abbreviation: conc., concentration; PGZ, Pioglitazone; PLGA, Poly(D,L-lactic-co-glycolic acid); MES, 4-Morpholineethanesulfonic acid; PVA, polyvinyl alcohol; DSPE-PEG, 1,2-distearoyl-sn-glycero-3-phosphoethanolamine-N-[amino(polyethylene glycol)-2000] Carboxylic Acid; DLS, Dynamic Light Scattering; HPLC, High-performance liquid chromatography. Created with BioRender.com.

In this regard, the single emulsification-solvent evaporation (A) or nanoprecipitation (B) methods were suggested as the best formulation methods for encapsulating hydrophobic drugs. In the single emulsification-solvent evaporation technique, first described by Vanderhoff et al. in 1979 [28], the nanoparticles are formed in two steps. A polymer solution is first prepared in a water-immiscible volatile organic solvent. A colloidal suspension of micelles is then formed by adding an emulsifier aqueous solution and next vigorously mixing the two phases through an ultrasonic homogenizer; the nanoparticle suspension is finally obtained by evaporation of the solvent. Lipid stabilizer, like 1,2-Distearoyl-sn-glycero-3-phosphoethanolamine (DSPE), are usually included in this method (Figure 1A) [29]. Recently, Shi et al. reported that the functionalization of a part of these lipids with Poly(ethylene glycol) (PEG) plays an important role in the biophysical and chemical properties (e.g., final size and stability) of nanoparticles [30]. Moreover, Takayama et al. reported that the phosphate group of DSPE-PEG is involved in the intermolecular interaction of the nanoparticles, notably affecting the physical properties and structure of the particles along with improving the bioavailability by prolonging the circulation time in the body [31]. On the other hand, the nanoprecipitation technique, firstly described by Fessi et al. in 1989 [32], is based on dropping a volatile water-miscible organic solvent containing the polymer in an aqueous solution. A colloidal nanoparticles suspension is formed under slow-stirring (Figure 1B). Therefore, unlike the first technique, in which a strong surfactant, such as DSPE-PEG, is necessary to stabilize the emulsion, a strong surfactant is not necessary in the case of nanoprecipitation, and the formed suspension can be stabilized by a mild amphiphilic compound, such as PVA. This method is usually simple, rapid, and produces particles with low PDI values [33]. Once the colloidal nanosuspension is synthetized, both synthetic strategies require a purification step (Figure 1C), usually followed by characterization, e.g., by Dynamic Light Scattering (DLS) and/or High-performance liquid chromatography (HPLC) (Figure 1D).

Over the last few decades, both single emulsification-solvent evaporation (A) or nanoprecipitation (B) methods were employed to create water-stable PLGA nanospheres for pioglitazone delivery against different disorders. For instance, Kanemaru M. et al. synthetized PLGA nanoparticles via the single emulsion-solvent evaporation method for local delivery of pioglitazone to attenuate skin fibrosis in model mice; with the same synthetic methodology, Woo et al. encapsulated pioglitazone within PLGA nanospheres for the treatment of Type 2 diabetes and, similarly, Laddha U. D. et al. prepared PGZ-PLGA nanoparticles for the treatment of diabetic retinopathy [34,35,36]. Conversely, other groups selected the nanoprecipitation method as ideal to develop PGZ-NPs. Lewis D. R. et al. developed pioglitazone-loaded nanoparticles via nanoprecipitation to inhibit macrophage activation and decreasing atherosclerotic plaque rupture; Silva-Abreu M. et al. showed that PGZ-NPs obtained by the solvent displacement (or nanoprecipitation) technique have in-vivo anti-inflammatory efficacy for preventing ocular inflammation and that such PLGA nanocarriers functionalized by PEG moieties can potentially cross the brain endothelium and have positive effects in the treatment of a mouse model of Alzheimer’s disease [37,38,39]. As demonstrated also in these works, these approaches offer nanoparticles with particularly enhanced biocompatibility, tissue permeability, and drug release control, along with facilitated targeted drug delivery and a prolonged circulation time thanks to avoiding rapid renal clearance. Thus, both methods are suitable for pioglitazone encapsulation, but which one is the most promising method is still not clear; furthermore, a reliable synthetic formulation for adapting the reported synthetic methodologies for the encapsulation of many compounds, like PGZ, is still lacking and there are still many obstacles to overcome in order to realize their clinical potential.

The aim of the current study is (i) to clarify which is the ideal method for encapsulating PGZ within PLGA nanoparticle and (ii) to establish a simple, rapid, and reliable synthetic formulation for this. In the first part of this work, we investigated the impact of several formulation parameters on the physicochemical properties of the nanoparticles, such as size, polydispersity index (PDI), and zeta potential (ζ_p_); amongst these parameters, we considered: (i) polymer concentration, (ii) surfactant or additive presence, (iii) buffer pH and concentration, (iv) phases ratio, (v) injection flux, (vi) sonication amplitude and time. Our optimization aimed to develop PGZ-loaded NPs with PDI closest as possible to zero, in order to have a monodisperse NP family and therefore more reproducible outcomes, the highest possible entrapment (or encapsulation) efficiency (EE%) and drug loading (DL%), in order to encapsulate the highest amount of PGZ and reduce the dose and frequency of the administration, along with a sufficiently high ζ_p_ (in absolute value), which helps nanoparticle stability. Moreover, an appropriate particle size for our PGZ-loaded NPs should range between 200–300 nm. Nanoparticles with these diameters have the main advantage of circumventing the reticuloendothelial system uptake, thus increasing PGZ bioavailability, and achieving a high atheroma targeting, thus reducing PGZ systemic side effects [40,41]. Smaller particles (<200 nm) have a greater surface area/size ratio than bigger ones, and thus a greater aggregative phenomena tendency. The authors reported that these NPs might circulate with no specific target: it could become a disadvantage (e.g., they could cross the blood–brain barrier also when this is unwanted) or advantage (e.g., they could cross the gaps in blood vessels supplying tumorous cells without significant penetration into healthy tissues). On the contrary, larger particles (>300 nm) have a very low bioavailability, as they might trigger the complement system and could be rapidly removed from the blood stream [42]. Once optimized, the best formulations for each method were compared concerning entrapment efficiency, drug loading, drug release and stability rates.

## 2. Results and Discussion

Several nanoparticle platforms to encapsulate PGZ were developed via single emulsification-solvent evaporation or nanoprecipitation approaches. A screen of 22 nanoparticle formulations was done in order to achieve the optimum over size, PDI, ζ_p_, EE% and DL%, which have noteworthy implications on PGZ pharmacokinetics, such as cellular uptake and metabolism. In this section, we shall present a rational optimization of these parameters for the single emulsification-solvent evaporation method (Section 2.1) and for the nanoprecipitation formulation method (Section 2.2). Then, a comparison between the physicochemical properties of the two designated synthetic methodologies is discussed (Section 2.3).

For our synthetic processes, since lower PLGA molecular weight implies lower PLGA hydrophobicity and probably a worse interaction with DSPE-PEG, we determined that PLGA at molecular weight (Mw) 38,000–54,000 Da (from here on, PLGA*) was more suitable than PLGA with Mw 24,000–38,000 Da (from here on, just PLGA) within the emulsion-evaporation approach, and vice versa within the nanoprecipitation method. Indeed, using the lower-weight PLGA in single emulsification-solvent evaporation method, polymer strands precipitated within the solution, meaning that NPs were not obtained. Moreover, as discussed by Chen et al., the organic solvent nature and volume and the drug solubility in this phase influence the NPs size and the NPs polydispersity index [43]. In the single emulsification-solvent evaporation approach, chloroform allows an optimal PLGA* dissolution and it forms two phases with water. In the nanoprecipitation approach, acetone represents an excellent choice, since it can completely dissolve the polymer and it is fully miscible with water. A higher water miscibility allows a higher efficiency in solvent diffusion and polymer dispersion into water, obtaining smaller sizes and PDI.

### 2.1. A Rational Optimization of the Single Emulsification-Solvent Evaporation Formulation Parameters

In the single emulsification-solvent evaporation method, NPs are not formed instantaneously: some hours are necessary, also because the organic solvent has to evaporate. To minimize the loss of PGZ due to a diffusion from the dispersed phase (organic solution) to the dispersing phase (aqueous solution), we carried out preliminary tests to optimize the pH of the aqueous solution using a buffered system. Since the PGZ is a weak acid (pKa 5.19), the solubility can be reduced working with an acid dispersing phase [44]. From these first experiments, an optimal pH of 6.2 was found, whilst NPs were not obtained at a lower pH. Accordingly, an MES buffer (pKa 6.15) was chosen as buffer system at pH 6.2.

**Figure 2:**
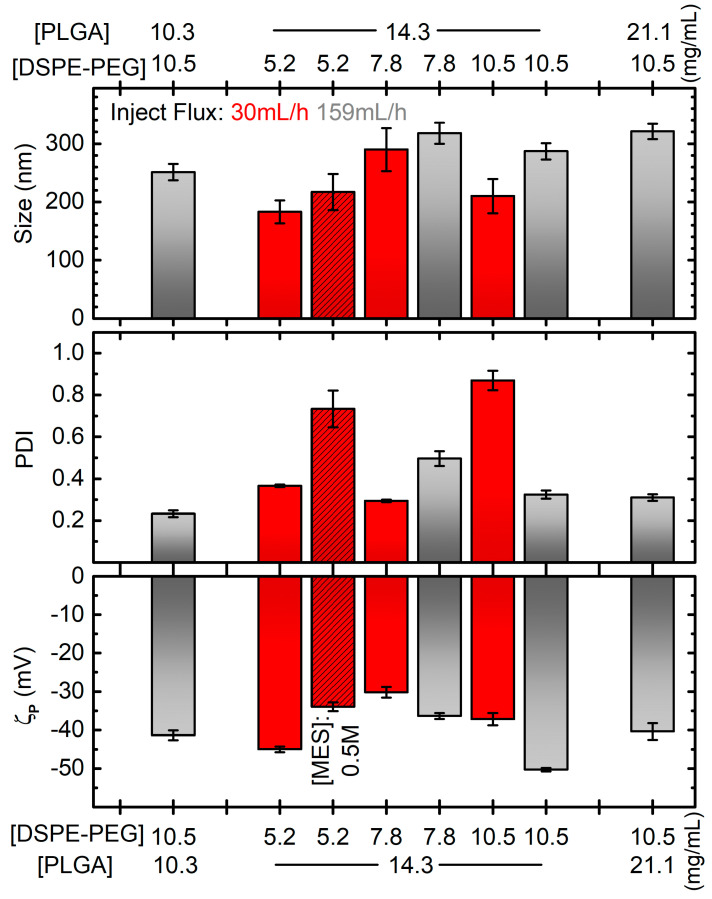
Diameter (**top**), PDI (**middle**) and zeta potential (ζ_p_, **bottom**) of PGZ-NPs developed by single emulsification-solvent evaporation method as a function of DSPE-PEG and PLGA concentrations and different parameters: inject flux 159 mL/h for grey bars, 30 mL/h for red bars; MES concentration of 0.5 M for dashed bar, 0.1 M in all the other cases. All the other parameters are like the bold ones in Table 1, and in particular the “reference” formulation corresponds to the grey bar at [PLGA] = 14.3 mg/mL and [DSPE-PEG] = 10.5 mg/mL. Error bars are standard errors. Horizontal grey dotted lines correspond to the values for the formulation considered the best compromise in terms of the plotted quantities.

Following the protocols found in the literature and preliminary works in our laboratory, the initial PLGA* and DSPE-PEG concentrations were set at 14.3 mg/mL and 5.2 mg/mL, respectively [45,46,47]. Moreover, we observed that using the maximum sonication amplitude did not produce suitable NPs: using 100% sonication power with all the other standard parameters as in Table 1 (or even changing the DSPE-PEG concentration to 7.8 mg/mL), DLS experiments showed the presence of NPs of diameters around 300 (260) nm, but the PDI was not so good, around 0.88 (0.40), and most importantly, the ζ_p_ was very low in absolute value, being equal to −0.13 ± 0.17 mV (−13 ± 8 mV; compare all these data with the ones reported in Figure 2 for NPs obtained at a sonication power of 80%). Although there are reports that an increase in the power of sonication and injection flux rate reduces the size of the emulsion droplets, an excessive shear stress could destroy the particulate system or forms several particle populations [48]. Hence, for our experiments, the sonication amplitude was decreased at 80% and the injection flux was initially set at 30 mL/h.

After these preliminary decisions, other parameters were evaluated, such as the effect of MES, DSPE-PEG and PLGA* concentration along with the influence of the injection flux rate on the diameter, PDI, and ζ_p_ of nanoparticles (Figure 2).

Firstly, two MES concentration values (0.1 M and 0.5 M) were studied. As reported in Figure 2, adequate size (around 200 nm) and satisfactory |ζ_p_| (greater than 30 mV) values were found with both concentrations. On the contrary, a narrower polydisperse population was found with 0.1 M (PDI around 0.3) than 0.5 M (PDI around 0.7). The lower viscosity and the lower amounts of salts in the 0.1 M MES solution probably allow a better homogenization during sonication. Hence, a 0.1 M buffer concentration was employed for our following synthesis.

Then, the DSPE-PEG concentration effect was evaluated. As discussed in the introduction, PEGylated lipid coating has a pivotal role in improving the physicochemical properties of nanoparticles. For our single emulsification-solvent evaporation synthesis, 5.2 mg/mL, 7.8 mg/mL or 10.5 mg/mL of DSPE-PEG were employed. As we can observe from the red non-dashed bars in Figure 2, the average diameter, PDI and ζ_p_ were very variable without a clear correlation, and with the best formulation considering PDI being the worse considering ζ_p_. Moreover, we observed precipitates during DLS measurements for every sample, indicating that most of the reagents had probably not reacted. We hypothesized that the addition of the organic phase to the dispersant phase was not rapid enough to obtain stable particles. Thus, the syntheses were repeated with the injection flow of the dispersed (organic) phase increased to 159 mL/h. Except for the unexpected result for the formulation prepared with 5.2 mg/mL of DSPE-PEG, which showed macroscopic white aggregate (DLS measurements not evaluated), stable nanoparticles without precipitate were obtained. DLS size measurements showed no significant differences using DSPE-PEG of 7.8 mg/mL and 10.5 mg/mL, with diameters around 270 nm. Conversely, the PDI improved from 0.5 to 0.3 by increasing DSPE-PEG amount. A similar trend was found for ζ_p_, with values from −36 to −50 mV. Hence, we demonstrated that the injection flux improvement could better stabilize the nanoparticle suspension and that the PEGylated lipid concentration plays a key role in the development of uniform NPs. From here on, 10.5 mg/mL of DSPE-PEG amount was employed as optimal for encapsulating our drug.

Next, the effect of PLGA* concentration over the diameter, PDI and ζ_p_ of PGZ-loaded nanoparticles were assessed, while the rest of the conditions and procedures were maintained constant. One lower (10.3 mg/mL) and one higher (21.1 mg/mL) PLGA* concentrations were assessed in addition to the one used in the syntheses discussed above (14.3 mg/mL). Observing the grey bars (DSPE-PEG concentration at 10.5 mg/mL) in Figure 2, there is a positive correlation between the PLGA* concentration and the size of the NPs, which increases from ~250 to ~320 nm for a polymer concentration increasing from 10.3 to 21.1 mg/mL. This is probably due to a greater quantity of PLGA* that had to be contained within the DPSE-PEG shell. The effect on PDI and ζ_p_ is much less pronounced and less clear, obtaining PDIs between 0.23 and 0.32 and and ζ_p_ between −50.3 mV and −40 mV. Despite the bigger ζ_p_ (in absolute values) for NPs obtained at the intermediate tested concentration of PLGA*, lower diameter size and PDI values were achieved with its lowest value. Hence, we deemed this last formulation to be more appropriate for our aim.

In summary, we found that the lowest assessed PLGA* concentration and the highest assessed DSPE-PEG concentration represent the best compromise to prevent aggregative phenomena and to develop, via the single emulsification-solvent evaporation method, a homogenous and stable PGZ-loaded PLGA nanoparticulate.

### 2.2. A Rational Optimization of the Nanoprecipitation Formulation Parameters

**Figure 3:**
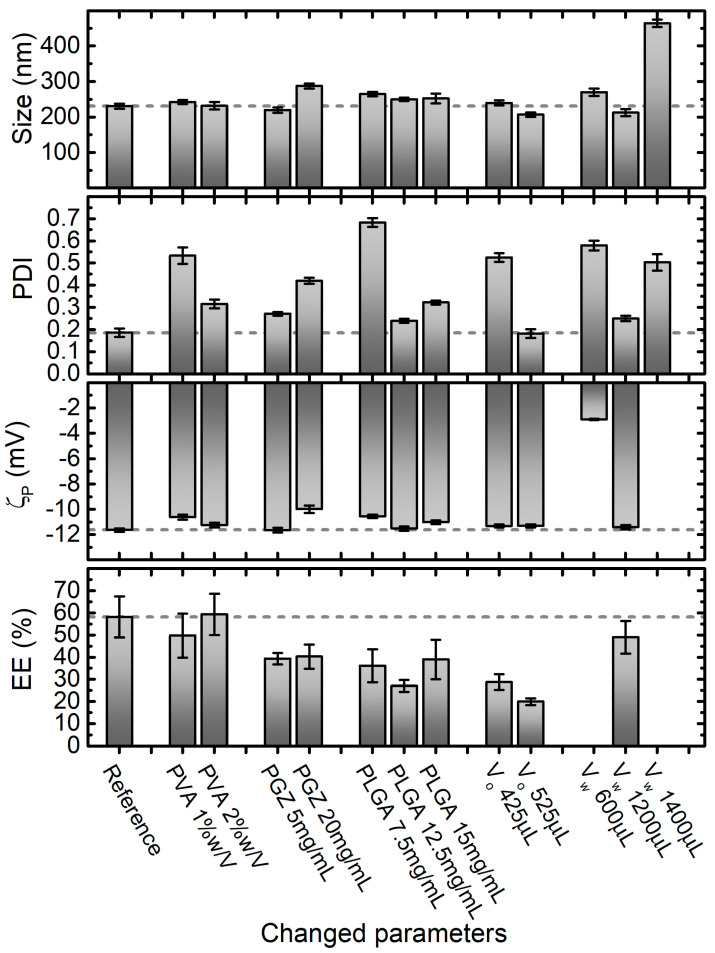
Effect of varying nanoprecipitation formulation parameters on PGZ-loaded NPs size, PDI, ζ_p_ and entrapment efficiency (EE%). Parameters in the Reference formulation are the bold ones in Table 1 (in particular, PVA 4% w/V, PGZ 10 mg/mL, PLGA 10 mg/mL, dispersant phase volume V_W_ 800 μL, and dispersed phase volume V_O_ 225 μL equal to V_W_/3.5). Error bars are standard errors. Dashed lines are at the values of size, PDI, ζ_p_ and EE% of the formulation that we identify as the best compromise in terms of these parameters, which in this case corresponds to the reference one.

The effect of PVA, PGZ and PLGA concentration along with the influence of the organic and the aqueous phase volumes over the diameter size, PDI, ζ_p_ and EE% of nanoparticles, are presented in Figure 3. Unlike the single emulsification-solvent evaporation, a slightly lower pH (6.0) could be used in the nanoprecipitation method with the aim of improving the PGZ encapsulation efficiency.

Stock aqueous solutions of PVA at 1%, 2%, and 4% were used for the preparation of NPs with the nanoprecipitation technique, obtaining final PVA concentrations within the aqueous phase of 0.5%, 1%, and 2%, respectively. In this range of PVA concentrations, no significant effect on the diameter size and ζ_p_ of PGZ-loaded NPs was observed, with diameters and ζ_p_ values around 235 nm and −11 mV, respectively, in all formulations. Inversely, the PDI value decreases approximately from 0.5 to 0.2 with the increase of PVA. This could be explained considering that, at low concentrations, PVA is not well adsorbed on the nanoparticle surface and is not able to stabilize the NPs. On the other hand, higher PVA concentrations are covering better the nanoparticle surface and, interacting by hydrogen and Van der Waals bonds with PLGA, can stabilize it. This is also supported by the ζ_p_ values slightly decreasing (increasing in absolute value) with the increase of PVA.

Moreover, the PLGA concentration showed a noteworthy effect, especially on PDI. Four concentrations of PLGA were used in the nanoparticle preparations by nanoprecipitation: 7.5 mg/mL, 10.0 mg/mL, 12.5 mg/mL and 15.0 mg/mL. In this range, nanoparticles with average diameters of 230–260 nm were obtained, indicating the 10 mg/mL formulation as optimal (231 ± 7 nm). The PLGA concentration plays a pivotal role in the development of uniform NPs. As reported in Figure 3, a PLGA concentration lower than a certain threshold (10 mg/mL in our case) is not satisfactory to form reasonable nanoparticles (PDI around 0.7). Instead, with PLGA concentrations higher than 10 mg/mL, NPs are formed but they have less uniform size, probably because all the polymer is not completely stabilized by PVA. On the contrary, ζ_p_ did not show big variations in the evaluated range of PLGA concentrations, obtaining ζ_p_ values around −11 mV, with the best value obtained again at the PLGA concentration of 10 mg/mL.

Next, three different organic phase volumes (V_O_) were employed to assess the four characteristics shown in Figure 3 for the nanoparticles obtained via the nanoprecipitation technique. 10.0 mg/mL, 18.9 mg/mL and 23.3 mg/mL of PGZ dissolved in 25 μL of DMF were mixed respectively with 200.0, 400.0 and 500 μL of acetone solution, containing PLGA at 10 mg/mL. In this way, we changed the volume but not the composition of the organic phase. Except for the unexpected result for the formulation prepared with 425.0 μL of Vo, which showed a dramatically higher PDI value (0.525 ± 0.0198), no significant effect on the diameter size and PDI of PGZ-loaded NPs was observed using a Vo of 225.0 and 525 μL, obtaining diameters around 230 nm and PDI values around 0.18 respectively. Similarly, ζ_p_ did not show variations in the evaluated range of Vo (average ζ_p_ = −11.4 mV). However, we noticed that the EE% decreased using higher Vo.

To further reduce the ratio between organic phase and aqueous phase and to avoid problems due to the solubility of the reagents (for the organic phase), we did not further reduce the organic phase, but we increased the aqueous phase. Four V_W_ were used in the nanoparticle preparations by nanoprecipitation, while keeping the MES buffer and PVA solution volumes ratio constant at 1:1. As depicted in Figure 3, the average diameter, PDI and ζ_p_ values were highly variable and only 800 μL and 1200 μL of Vw allowed to fabricate stable and homogeneous nanoparticles. The EE% of the 600 μL and 1400 μL formulation were not evaluated, because of the too-high PDI values. Changing the V_o_ and V_w_, we evaluated the NPs changes with the fraction volume of the organic phase. This parameter is important for the size optimization of the NPs: standard ratio between 1:3 to 1:10 are usually used [49]. With the reduction of the ratio between the organic phase and the aqueous phase, an increase in diffusion of the water-miscible organic solvent is obtained. The optimal ratio allows a fast diffusion, but is slow enough for the formation of the NPs. The best results in terms of EE% and PDI were achieved using 225 μL for the organic phase and 800 μL for the aqueous phase.

The final considered parameter was the concentration of PGZ in the organic phase (VO). The effects of 5 mg/mL, 10 mg/mL (Reference) and 20 mg/mL PGZ concentrations were evaluated, obtaining nanoparticles with increasing average diameters of 219 ± 7 nm, 231 ± 7 nm, and 287 ± 7 nm, respectively. In agreement with Hernández-Giottonini et al., the average diameter values of PGZ-loaded nanoparticles developed via nanoprecipitation method tend to increase as the PGZ concentration increase [33]. In an analogous way, the effects of PGZ concentration over the PDI and ζ_p_ of nanoparticles are reported. As reported in Figure 2D, the PDI at the lowest and highest PGZ concentration are higher than at 10 mg/mL, indicating a larger size distribution; the ζ_p_ values ranged between −10 and −12 mV in the studied range of concentrations, denoting low but uniform stability. The EE% shows a maximum at 10 mg/mL and lower values at 5 mg/mL and 20 mg/mL of PGZ. A lower EE% for the 5 mg/mL concentration is anomalous; we could hypothesize that with the 10 mg/mL concentration, the diffusion of PGZ between the two phases is lower due to intermolecular interaction between different PGZ molecules. With a lower concentration, these interactions are less marked, causing faster diffusion and a lower EE%. On the other hand, for the 20 mg/mL formulation, the quantity of PGZ is too high to be encapsulated in the nanoparticles.

### 2.3. Comparison between the Two Syntheses

**Figure 4:**
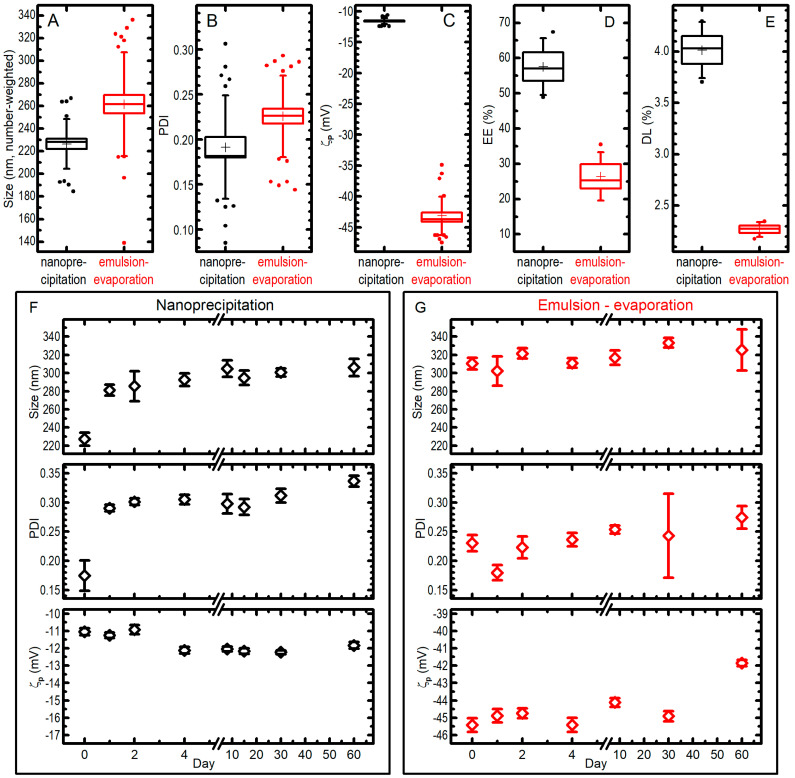
The best synthetic formulations for nanoprecipitation (black data) and single emulsion–solvent evaporation (red data) techniques were compared for size (**A**), PDI (**B**), ζ_p_ (**C**), entrapment efficiency EE% (**D**), drug loading DL% (**E**). In the box plots, boxes are standard errors (SEs), whiskers are standard deviations, lines are medians, crosses are averages, dots are outliers; all the data from different measurements (25 for nanoprecipitation, 31 for emulsion-evaporation for panels (**A**–**C**); 4 for panels (**D**,**E**)) on different syntheses (4 for nanoprecipitation and 5 for emulsion-evaporation in panels (**A**–**C**); 4 in panel (**D**) and 2 in panel (**E**) for both methods) are pooled together. Moreover, the behaviour of size, PDI, and ζ_p_ for a single preparation of PGZ-NPs via nanoprecipitation (**F**) or single emulsification-solvent evaporation (**G**) when kept at −20 °C for a different number of days is presented and compared with the ones of an aliquot of the fresh-prepared colloid (data at day 0); bars are SEs over 7–10 measurements.

The best synthetic formulation regarding size, PDI, ζ_p_ and EE% of nanoparticles were: (A) for single emulsification-solvent evaporation, 200.0 μL PLGA* (10.3 mg/mL) + 28.7 μL Pio (10.0 mg/mL) + 62.5 μL DSPE-PEG (10.5 mg/mL) + 2.0 mL MES (0.1 M, pH: 6.2), 159.0 mL/h injection flux rate, sonication time of 4.8 min with 80.0% of amplitude; and (B) for nanoprecipitation method, 200.0 μL PLGA (10.0 mg/mL) + 25.0 μL Pio (10.0 mg/mL) + 400.0 μL PVA (4.0%) + 400.0 μL MES (1.0 M, pH: 6.0), 30.0 mL/h injection flux rate. As depicted in Figure 4, these synthetic procedures were compared to establish the best PGZ-loaded PLGA NPs over the size, PDI, ζ_p_, EE%, DL%, and stability.

The nanoparticles developed via the nanoprecipitation method (B) had a mean diameter of 226 ± 22 nm (note: in this paragraph the uncertainties are standard deviations from all measurements pooled together; see Figure 4) and a very narrow size distribution (PDI = 0.19 ± 0.06), while the nanoparticles developed via the single emulsification-solvent evaporation method (A) had a higher mean diameter (262 ± 46 nm) and a significantly (at 0.05 level) larger size distribution with a PDI of 0.23 ± 0.05 (Figure 4A,B). The ζ_p_ measured for the nanoprecipitation technique showed an average value of −11.6 ± 0.5 mV, whilst ζ_p_ values for the single emulsification-solvent evaporation technique showed a more negative average value of −43.2 ± 3.1 mV (Figure 4C); we attribute this to the DSPE-PEG that is localized on the nanoparticle surface, which donates a higher charge (in absolute value) with respect to a simple PLGA nanoparticle. The EE% values of PGZ-loaded NPs were 58 ± 8% and 26 ± 7% for the nanoprecipitation and single emulsification-solvent evaporation techniques, respectively (Figure 4D). Instead, DL% were 4.01 ± 0.27% and 2.27 ± 0.07% (results from two independent syntheses) for the nanoprecipitation and single emulsification-solvent evaporation techniques, respectively (Figure 4E). These values strongly depend on the type of polymeric matrix, in particular on its molecular weight. The nanoprecipitation method presents a higher EE%, most likely because a higher velocity of nanoparticle formation causes a minor loss in PGZ due to diffusion in the aqueous phase, as described above.

Concerning the storage stability study, PGZ-loaded NP samples were stored at −20 °C and the nanoparticles characteristics were monitored over 60 days by DLS (Figure 4F,G). For the nanoprecipitation technique, the stability assay showed a certain propensity towards aggregation during the freezing process: NP diameters (number-weighted) went from 227 ± 22 nm to 306 ± 28 nm in 60 days (uncertainties in this paragraph are SDs from 7–10 repeated measures on a single batch of NPs). It must be noted, however, that the biggest change happens already at the first freezing–thawing cycle, while the dimensions increases only slightly in the following 59 days of observation for frozen samples. The PDI showed a trend similar to the one observed for the size, i.e., it was possible to notice an initial increase, passing from 0.17 ± 0.08 in day 0 to 0.29 ± 0.02 in day 1, due to the aggregation during the freezing and thawing process, and only a slow trend towards higher values after that. As far as ζ_p_ is concerned, no dramatic differences were highlighted over the 60 days. On the other hand, PGZ-loaded NPs obtained via the single emulsification–solvent evaporation method did not show important changes over freezing and time in 60 days: in the batch used in this experiments, mean (number-weighted) diameters went from 310 ± 20 to 325 ± 71 nm after staying frozen for 60 days; the size distribution and the ζ_p_ remained similar, reaching a PDI of 0.27 ± 0.06 and a ζ_p_ of −41.9 ± 0.6 mV after 60 days. We could expect the enhanced stability of the DSPE-PEGylated nanoparticles (single emulsification-solvent evaporation method), because of the more negative ζ_p_ due to the negatively charged DSPE-PEG (both from the phosphate group and the carboxylic acid). However, size and PDI of the two formulations remain comparable also after storage, and the significantly better EE% and DL% of NPs obtained by nanoprecipitation make them more suitable for a PGZ formulation.

Finally, the costs of both syntheses were calculated, taking into account the EE%. In order to encapsulate 0.145 mg of PGZ, reagents’ cost for the NPs obtained by nanoprecipitation and by the emulsion–solvent evaporation technique were 0.40 Euro and 7.22 Euro for a total NPs weight of 3.6 mg and 6.3 mg, respectively (Table 2). In addition, the emulsion–solvent evaporation method is more time-consuming, which in turn causes a further increase in the production costs. This showed the clear convenience of an encapsulation carried out by nanoprecipitation.

The best synthetic formulations and corresponding parameters are summarized in Table 3.

### 2.4. Release Assay

**Figure 5:**
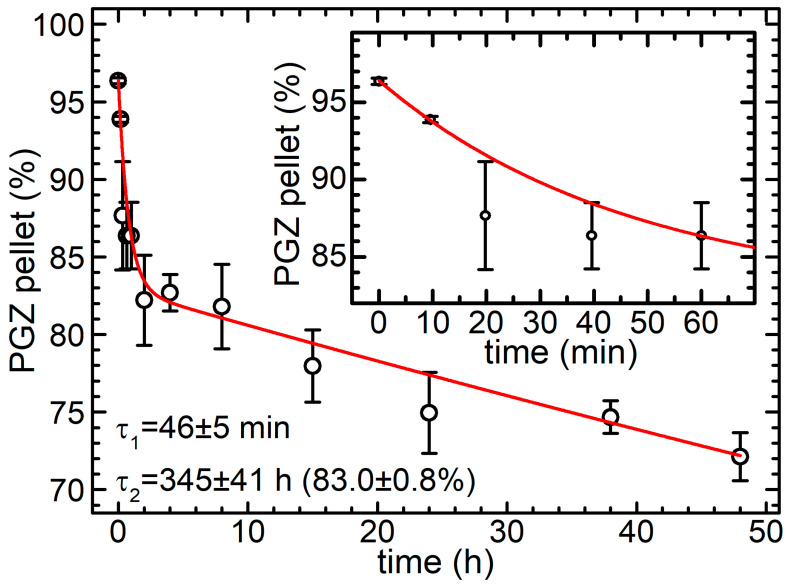
Percentage of PGZ remained inside PGZ-loaded PLGA NPs (developed via nanoprecipitation method) in phosphate-buffered saline (PBS) buffer at 37 °C as a function of time; error bars are standard deviations (n = 2), red line is a fit with a double-exponential decay to 0, the inset is a zoom of the graph in the first 70 min.

In order to control that the PGZ level in the media is within the suitable therapeutic window, it is important to examine drug release kinetics. Drug release from a polymeric NP can occur with three main mechanisms: (i) desorption of adsorbed/bounded substance on the surface; (ii) diffusion through the nanosphere matrix and/or through the nanocapsule wall; and (iii) erosion of the nanoparticle matrix [50]. We measured the percentage of drug remaining inside our PGZ-loaded PLGA NPs (obtained through nanoprecipitation) in a phosphate-buffered saline (PBS) buffer at 37 °C. The results are shown in Figure 5. PGZ release was followed for 48 h, and two stages were observed: an initial burst release followed by a slower phase. In order to be more quantitative, the data were fitted with a double exponential decay model; this indicated that 3.6 ± 0.5% of PGZ was already out of the NPs at the beginning of the experiment, and an additional 13.4 ± 0.8% was released with a halving time of 31.7 ± 3.4 min. As a result, ~17% of the PGZ is released within the first two hours. The remaining PGZ is then released slowly with a halving time of 239 ± 28 h, i.e., approximately 10 days. The plausible explanation is that part of the PGZ was loosely incorporated within the outer layer of the nanoparticles and was promptly released, whilst another part was incorporated more deeply, within the core of the nanoparticle. The instantaneous release of PGZ (3.6 ± 0.5%) is probably due to a desorption mechanism of the drug adsorbed on the nanoparticle surface, the following fast release is probably due to diffusion processes for molecules in less packed zones of the polymer matrix closer to the surface, and the final slower release could be caused by a slower reconfiguration of the polymer matrix, up to the point of erosion or hydrolyses of the PLGA polymer. The fact that only 17% of the encapsulated PGZ was released into PBS during the first two hours, with a much slower release of the remaining part, confirms the stability of our nanoconstructs, at least in PBS, and could be crucial for a pharmacological treatment, allowing enhancing the bioavailability of the drug and consequently expanding the therapeutic window.

## 3. Materials and Methods

### 3.1. Materials

**Scheme 1:**
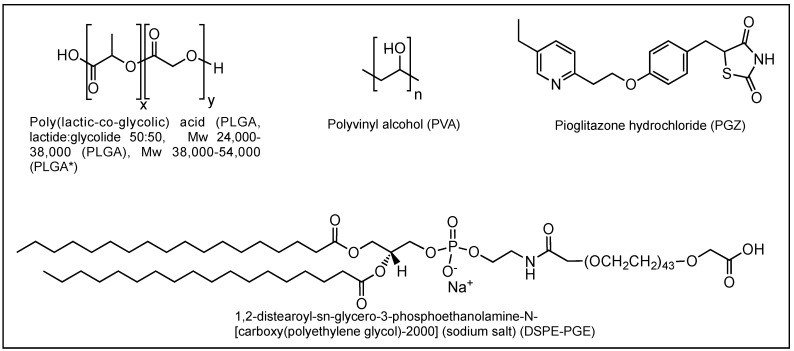
Chemical structure of poly(lactic-co-glycolic acid) (PLGA), polyvinyl alcohol (PVA), pioglitazone hydrochloride (PGZ) and 1,2-distearoyl-sn-glycero-3-phosphoethanolamine-N-[amino(polyethylene glycol)-2000] (DSPE-PEG).

All chemical reagents were purchased from Aldrich (Saint Quentin Fallavier, France) or Acros (Noisy-Le-Grand, France). Pioglitazone (PGZ), Resomer^®^ RG 503 H, PLGA acid terminated (lactide:glycolide 50:50, Mw 24,000–38,000, referred to as PLGA), Resomer^®^ RG 504 H, PLGA acid terminated (lactide:glycolide 50:50, Mw 38,000–54,000, referred to as PLGA*), D-(+)-Trehalose dihydrate, 4-Morpholineethanesulfonic acid (MES), polyvinyl alcohol (87–89% hydrolysed, Mw approx. 18,000, PVA) and all other chemicals were purchased from Merck KgaA (Darmstadt, Germany) and were used without further purification. 1,2-distearoyl-sn-glycero-3-phosphoethanolamine-N-[amino(polyethylene glycol)-2000] (DSPE-PEG(2000)) Carboxylic Acid was purchased from Avanti Polar Lipids (Alabama, USA) and was used without further purification. Ultrapure water was generated in-house using a MilliQ plus System (Merck KgaA, Darmstadt, Germany). Chemical structure of PLGA, PVA, PGZ and DSPE-PEG are reported in Scheme 1. The nanoparticles were freeze-dried with a Alpha 2-4 LSC lyophilizer (Christ, Osterode am Harz, Germany).

### 3.2. Nanoparticles Synthesis via Single Emulsification-Solvent Evaporation Method (A)

28.7 μL of DMF (N,N-Dimethylformamide) containing PGZ was mixed with an organic solution containing different concentrations of PLGA* in chloroform and then this “dispersed phase” was injected into a dispersant phase formed by mixing MES water-buffer and an ethanol solution of DSPE-PEG. Table 1 presents all the parameters investigated independently. The injection was performed with a pump 11 Elite Infusion/Withdrawal Programmable Single Syringe (Harvard Apparatus, Holliston, MA, USA) operated at 30 or 159 mL/h. The mixture was then emulsified while in an ice bath for 4.5 min at 80% amplitude (90 mm probe) using an ultrasonic homogeniser, SONOPULS (VWR International, Avantor, Milan, Italy), then the organic solvent evaporated under magnetic stirring at 400 rpm for 4 h at room temperature. NPs were washed by three centrifugation cycles using a Heraeus Pico^TM^ 17 microcentrifuge (Thermo Fisher Scientific, Waltham, MA, USA) operated at 8000× *g* (9100 rpm) for 5 min, discarding supernatant and resuspending the pelleted nanoparticles in MES buffer. The pellet was finally suspended in 200 μL of a trehalose cryoprotectant water solution (10 mg/mL) and stored at −20 °C. Experiments were performed in triplicate.

### 3.3. Nanoparticles Synthesis via Nanoprecipitation Method (B)

25.0 μL of DMF containing pioglitazone was mixed with an organic solution containing different concentrations of PLGA in acetone and then injected at 30 mL/h into a mix of MES buffer and PVA aqueous solution. Table 1 presents all the parameters investigated independently. The suspension was kept under magnetic stirring at 400 rpm during injection and for additional 10 min. Then, NPs were washed by three centrifugation cycles at 8000× *g* (9100 rpm) for 5 min, resuspending the pelleted nanoparticles in MES buffer and, after the last centrifuge cycle, in 200 μL of a trehalose cryoprotectant solution (10 mg/mL). Samples were stored at −20 °C.

### 3.4. High Performance Liquid Chromatography (Hplc) Method

Analytical RP-HPLC was performed on a Shimadzu Nexera UHPLC, equipped with a Shimadzu SPD-M20A UV/visible detector. Analyses were carried out at 0.80 mL/min on a Phenomenex column (Kinetex XB-C18, 5 μm, 100 × 2.1 mm) with UV monitoring at 270 nm, using a 15 mM ammonium acetate buffer with a pH of 8.0 (Eluent A) and an Acetonitrile:Eluent A 95:5 mixture (Eluent B). The chromatographic analysis was done with a linear gradient, starting from 35.0% of eluent B. The quantification of PGZ was performed with an external calibration curve.

### 3.5. Nanoparticle Size, Polydispersity Index and Zeta Potential Measurements

Nanoparticle size, PDI, and ζ_p_ were measured by dynamic light scattering (DLS) using a Zetasizer Nano ZS equipment (Malvern Instruments Ltd., Worcestershire, UK). A 100-µL aliquot of each trehalose-dissolved sample was diluted 1:54 in trehalose solution (10 mg/mL) and analysed. The nanoparticle size measurements were made at a fixed light scattering angle of 90°. The reported values refer to the number-weighted mean particles size distribution (unless otherwise stated). Each sample was measured at least six times for size analysis with a quartz cuvette (DTS2145). The reported ζ_p_ of each sample was measured each with a disposable folded capillary cell (DTS1070). For the analyses, NPs refraction index was 1.590 (absorption 0.010) and water was used as the dispersant (25 °C, viscosity 0.8872 cP, refractive index 1.330, dielectric constant 78.5). Size averages and zeta potentials were obtained from at least three independent experiments. In this work, we will consider mostly number-weighted distributions (%$N_{a}$), especially for sizes. %$N_{a}$ distributions are actually obtained from the intensity-weighted ones ($\%I_{a}$), which are the ones obtained by fitting the dynamic light scattering signals, by considering that nanoparticles have scattering intensities proportional to the sixth power of their radii: e.g., considering a distribution of particles having only two sizes a and b, the two distributions are given by

(1) $$\%I_{a}=\frac{a^{6}N_{a}*100}{N_{a}a^{6}+N_{b}b^{6}}$$

(2) $$\%N_{a}=\frac{N_{a}*100}{N_{a}+N_{b}}$$

where $N_{x}$ represents the population of molecules with size x (x=a,b) [51].

### 3.6. Drug Loading (DL%) and Entrapment Efficiency (EE%) Measurements

In order to determine the quantity of PGZ in a sample, a 140-µL aliquot of each trehalose-dissolved sample was sonicated and lyophilized overnight. The samples were weighted and the weight corrected for the trehalose content. The freeze-dried samples were then dissolved in a mixture of 80 μL DMSO, 130 μL buffer A, 130 μL buffer B solution, selected for the validated HPLC method. A calibration curve was prepared using a mixture of 50 μL DMF, 60 μL buffer A, 140 μL buffer B solution ranging from a concentration of 4 to 1720 μg/mL of PGZ (R^2^ = 0.999). Experiments were performed by triplicate.

The drug loading percentage (DL%) and entrapment efficiency percentage (EE%) values were calculated as follows:

(3) $$\mathrm{DL}\%=\frac{\mathrm{Weight}\;\mathrm{of}\;\mathrm{drug}\;\mathrm{in}\;\mathrm{the}\;\mathrm{sample}\;\times 100}{\mathrm{Weight}\;\mathrm{of}\;\mathrm{drug}\;\mathrm{loaded}\;\mathrm{nanoparticles}}\;$$

(4) $$\mathrm{EE}\%=\frac{\mathrm{Weight}\;\mathrm{of}\;\mathrm{drug}\;\mathrm{in}\;\mathrm{the}\;\mathrm{sample}\;\times 100}{\mathrm{Weight}\;\mathrm{of}\;\mathrm{drug}\;\mathrm{used}\;\mathrm{in}\;\mathrm{the}\;\mathrm{synthesis}}$$

### 3.7. Stability of Frozen Aliquots

A 100 µL aliquot of each freeze-dried trehalose dissolved sample was withdrawn at various time intervals and diluted in 1500 µL of an aqueous solution of trehalose. Nanoparticle size, PDI and ζ_p_ were immediately evaluated as previously described.

### 3.8. Release Assay

A 100 µL aliquot of each trehalose dissolved sample was diluted in 1500 µL of phosphate-buffered saline (PBS) buffer and placed within a thermomixer comfort (Eppendorf, Milan, Italy) operated at 900 rpm and 37 °C. The samples were withdrawn at different time points (time 0 means few seconds of mixing) and centrifuged at 8000× *g* (9100 rpm) for 10 min. The pellets and supernatants were finally dried using the rotational-vacuum-concentrator RVC 2–18 CD plus (Martin Christ Gefriertrocknungsanlagen GmbH, Osterode am Harz, Germany) for 4 h and dissolved in a mixture of 40 μL DMSO, 80 μL buffer A, 80 μL buffer B solution, selected for the validated HPLC method, with calibration curve obtained as described in Section 3.6. The 100% corresponds to the total amount of PGZ in each sample. Experiments were performed in duplicate.

The percentage of PGZ remained within the NPs (*PGZ_NP_*) as a function of time has been fitted with a double exponential decay with asymptote at 0%:

(5) $$PGZ_{NP}=A_{1}e^{-\frac{t}{\tau_{1}}}+A_{2}e^{-\frac{t}{\tau_{2}}}$$

### 3.9. Data Analysis

Data analysis and graphs preparation were carried out using Origin 9 (ver. 9.0) and Prism (7.0). The results obtained from size, PDI, ζ_p_, drug-loading, entrapment efficiency, stability and release experiments are shown as mean ± standard error (SE).

## 4. Conclusions and Future Perspectives

In recent years, substantial studies towards novel strategies to overcome the several intrinsic limitations of drugs have been done. The improvements in manufacturing processes of nanosystems, which can improve the pharmacokinetics of drugs and potentially deliver therapeutic payloads to target cells involved in pathophysiological processes, has formed a cornerstone of these research efforts. However, synthetic protocols, e.g., of polymeric nanoparticles, still need to be optimized for making their production cost-effective, simple, rapid and reliable. Most of the strategies towards this purpose are still in the developmental stage and challenges need to be taken care of.

This article specially attempted to capture the state-of-the-art in pioglitazone encapsulation using PLGA nanoparticles. Having worked with two common strategies for the synthesis of nanomaterials, we conclude that the nanoprecipitation method is more suitable for our goals, providing better results in terms of size, PDI, EE%, DL%, cost, and processing time. The NPs obtained through the single emulsion–solvent evaporation method gave better results only in the stability assay, because those obtained by nanoprecipitation showed a tendency to aggregate during the freezing and thawing process. This behaviour is certainly linked to the less negative value of ζ_p_ for NPs obtained with the nanoprecipitation method. However, it is worth mentioning that their average diameter still remains in the desired range (between 200 and 300 nm) even after aggregation.

A general optimal synthetic method does not exist, but the structure of the NPs must be tuned depending on the chemical-physical characteristics of the encapsulated drug. In fact, the emulsion–solvent evaporation method is most probably the best choice for drugs with higher logP values. In these cases, higher hydrophobicity reduces the solubility in the continuous phase (aqueous phase), resulting in a greater EE%, and we have shown that stability of NPs is conserved during the freezing–thawing process.

As a possible future perspective, we are, however, confident that the stability of our NPs obtained by nanoprecipitation can be improved, for instance, by (i) optimising the cryoprotectant solution, or (ii) using in part PEGylated PLGA to reduce the aggregation tendency without substantially changing the NPs characteristics [52].

Next, we are currently working on the development of an efficient targeted delivery system. Since our nanocarrier is not fluorescent per se, its visualization in a biological environment requires labelling with fluorophores. In this regard, we consider of utmost importance the synthesis of NPs as imaging agents, labelled with the desired dye and with a suitable size, brightness and stability; this requires a selection of the optimal strategy for the preparation of the labelled particles, e.g., physical adsorption or covalent binding of the dye to the PLGA polymer [53,54]. Then, this nanosystem will be engineered for delivering drugs to particular tissues, e.g., by targeting proteins that are overexpressed or specific to malignant cells [55,56]. In this regard, biological investigations of the effects of the nanoarchitectures on the target cells represent an important step for predicting their consequences in more physiological environments, like in-vivo. For instance, once PGZ-loaded NPs cellular uptake capacity will be confirmed, our purpose will be to demonstrate their anti-atherosclerotic effect by inhibiting macrophage activation primary in vitro and then in vivo; another possible experiment would be the analysis of the effect on cell metabolism of PGZ versus PGZ-loaded nanoparticles.

Finally, particular attention should be paid to translating these synthetic methods into microfluidic systems, since this allows a continuous production of NPs and reduces batch-to-batch variability, offering an easier standardization of the parameters [57]. The resultant higher quality of produced nanoparticles is expected to accelerate the research of nanoparticles for biomedical applications, allowing more nanosystems to be able to reach clinical trial phases and hopefully be approved by regulatory agencies.

## Figures and Tables

**Table 1:** Parameters used in pioglitazone-loaded PLGA nanoparticles synthesis and purification; in italic, the parameters found for the best synthesis; in bold the standard ones, while in different syntheses mostly only one of the other multi-valued parameters was changed. The concentration values refer to the concentration within the used stock solution, not to the one in the final solution.

	Single Emulsification-Solvent Evaporation Method (A)	Nanoprecipitation Method (B)
PLGA concentration (mg/mL)	---	7.5, ***10***, 12.5, 15
PLGA* concentration (mg/mL)	*10.3*, **14.3**, 21.1	---
PGZ concentration (mg/mL)	***10***	5, ***10***, 20
Volume ratio PLGA:PGZ	***7.7:1***	***8:1***
Dispersed phase volume (μL)	***228.7***	***225***, 425, 525
Dispersant phase volume (μL)	***2062.5***	600, ***800***, 1200, 1400
MES concentration (mg/mL)	97.6, ***19.5***	***195.2***
pH of dispersant phase	***6.2***	***6.0***
PVA concentration (% w/V)	---	1, 2, ***4***
DSPE-PEG(2000) Carboxylic Acid concentration (mg/mL)	5.2, 7.8, ***10.5***	---
Dispersed:dispersant phases Ratio	***1:9***	1:1.5, 1:1.9, 1:2.66, ***1:3.5***, 1:5.3, 1:6.2
Stirring rate (rpm)	***400***	***400***
Stirring time (min)	***10***	***10***
Injection flux rate (mL/h)	30, ***159***	***30***
Sonication amplitude (%)	***80***, 100	---
Sonication time (min)	**4.8**	---
Centrifugation speed [rpm (× *g*)]	***9100 (8000)***	***9100 (8000)***
Centrifugation time (min)	***5***	***5***
Cryoprotectant concentration (mg/mL)	***10***	***10***

**Table 2:** Cost estimation summary for encapsulation of PGZ (0.145 mg) within PLGA nanoparticles via nanoprecipitation or emulsion–solvent evaporation methods. Costs reference period: 2021.

			Nanoprecipitation			Emulsion–Solvent Evaporation		
Reagent	Cost/Unit		Quantities Used for the Synthesis		Costs (€)	Quantities Used for the Synthesis		Costs (€)
DMF	155.-	€/1 L	25.0	µL	0.004	55.1	µL	0.0085
MilliQ water	13.6	€/1 L	2.60	mL	0.0354	11.9	mL	0.0054
PVA (4%)	211.-	€/500 g	48.0	mg	0.020			
MES (1 M)	501.-	€/500 g	234	mg	0.235			
MES (0.1 M)	501.-	€/500 g				224	mg	0.225
PGZ	34.1	€/50 g	0.250	mg	0.0002	0.551	mg	0.00038
Trehalose (10 mg/mL)	472.-	€/250 g	2.00	mg	0.004	3.84	mg	0.0072
PLGA	238.-	€/5 g	2.00	mg	0.095			
PLGA*	225.-	€/5 g				3.95	mg	0.178
Acetone	47.4	€/1 L	200	µL	0.0095			
Chloroform	107.-	€/1 L				384	µL	0.041
DSPE-PEG	268.-	€/50 mg				1.26	mg	6.75
Ethanol	39.6	€/500 mL				120	µL	0.0095
			**Sum (in Euro)**		**0.403**	**Sum (in Euro)**		**7.22**

**Table 3:** Summarized results on Pioglitazone-loaded PLGA nanoparticles synthesis; the best compromise formulations are considered.

Method	Dispersed Phase Conditions	Dispersant Phase Conditions	Other Synthetic Parameters	Size (nm)	PDI	ζ_p_ (mV)	EE (%)	DL (%)	Cost for Encapsulating 0.145 mg PGZ (€)
**Single emulsification-solvent evaporation (A)**	200.0 μL PLGA* (10.3 mg/mL) + 28.7 μL Pio (10.0 mg/mL)	62.5 μL DSPE-PEG (10.5 mg/mL) + 2.0 mL MES (0.1 M, pH: 6.2)	159.0 mL/h injection flux rate, sonication time of 4.8 min with 80.0% of amplitude	262 ± 46	0.23 ± 0.05	−43.2 ± 3.1	26 ± 7	2.27 ± 0.07	7.22
**Nanoprecipitation (B)**	200.0 μL PLGA (10.0 mg/mL) + 25.0 μL Pio (10.0 mg/mL)	400.0 μL PVA (4.0%) + 400.0 μL MES (1.0 M, pH: 6.0)	30.0 mL/h injection flux rate	226 ± 22	0.19 ± 0.06	−11.6 ± 0.5	58 ± 8	4.01 ± 0.27	0.403

## Data Availability

Data are contained within the article.
